# Early postoperative effects of cardiopulmonary bypass surgery on optic nerve head and peripapillary retinal structures: an OCT-A study

**DOI:** 10.1186/s12886-026-04830-9

**Published:** 2026-04-22

**Authors:** Mehmet Özbaş, Gülsüm Türkyılmaz, Sümeyye Selay Ersoy Şenel, Filiz Bakkal, Ali Aycan Kavala, Aslı Vural

**Affiliations:** 1 Department of Ophthalmology, University of Health Sciences, Bakırköy Dr. Sadi Konuk Training and Research Hospital, Zuhuratbaba Mah., Tevfik Sağlam Cad., Bakırköy, Istanbul, 34147 Turkey; 2 Department of Cardiovascular Surgery, University of Health Sciences, Bakırköy Dr. Sadi Konuk Training and Research Hospital, Istanbul, Turkey

**Keywords:** Cardiopulmonary bypass, Optic nerve head, Retinal nerve fiber layer, Optical coherence tomography angiography, Radial peripapillary capillary density

## Abstract

**Purpose:**

To quantitatively assess the early postoperative effects of cardiopulmonary bypass (CPB) surgery on optic nerve head (ONH) morphology, peripapillary retinal nerve fiber layer (PRNFL) thickness, and radial peripapillary capillary density (RPCD) utilizing optical coherence tomography angiography (OCT-A).

**Methods:**

A prospective observational study was conducted involving 40 eyes of 40 patients undergoing CPB surgery. Baseline (preoperative) measurements of ONH parameters, PRNFL thickness, and RPCD were acquired using OCT-A. Postoperative evaluations were performed at postoperative week 1, week 2, and month 1. Statistical analyses were performed using repeated measures analysis, with paired t-tests or Wilcoxon signed-rank tests applied for post hoc pairwise comparisons depending on data distribution.

**Results:**

A statistically significant but transient increase in PRNFL thickness was observed at postoperative week 1 and week 2 (*p* < 0.05), which returned to baseline by the first month. No significant differences were detected between preoperative and postoperative measurements concerning RPCD, cup-to-disc ratio, rim area, disc area, or cup volume.

**Conclusion:**

CPB surgery was associated with early but reversible changes in the ONH and peripapillary retinal structures in this study. OCT-A may serve as a useful non-invasive tool in the assessment of these transient changes. Longer-term follow-up studies are necessary to determine the sustained and permanent ocular effects of CPB surgery.

## Introduction

Postoperative visual loss or diminution, although rare, represents a serious complication following non-ocular surgeries, particularly those involving cardiac and spinal procedures [1–3]. Cardiopulmonary bypass (CPB) provides temporary circulatory and respiratory support via an extracorporeal circulation (ECC) system during complex surgical procedures. However, previous studies have reported various postoperative ocular complications associated with CPB surgery [3–5].

Hemodynamic and hematological changes associated with CPB, including the potential for microemboli, chorioretinal hypoperfusion, and localized hypoxia, may lead to ischemic optic neuropathy (ION), retinal nerve fiber damage, and retinal ischemia [6–7].

These changes may result in severe postoperative vision loss and other neuro-ophthalmic morbidities [8]. Although the incidence of ION subsequent to CPB surgery is relatively low [3–5], it is higher than non-cardiac surgical procedures [9]. The optic nerve head (ONH) is particularly vulnerable to hemodynamic fluctuations during CPB. Intraoperative factors such as profound hypotension, hypothermia, and activation of the complement system may contribute to ischemic changes in the optic nerve head [10].

This study aimed to evaluate early postoperative changes in optic nerve head (ONH) morphology, peripapillary retinal nerve fiber layer (PRNFL) thickness, and radial peripapillary capillary density (RPCD) using optical coherence tomography angiography (OCT-A), a non-invasive imaging modality that enables assessment of microvascular networks in the macular and optic disc regions. Currently, few studies have evaluated evaluating the impact of CPB surgery on the optic disc using OCT-A. In one such study, Gabija et al. [11] compared healthy individuals with patients undergoing CPB surgery. They demonstrated impaired retrobulbar circulation in CPB patients, which was associated with structural changes in the ONH and reduced vascular density in the optic disc.

The aim of this study was to evaluate the effects of CPB surgery on the ONH and peripapillary retinal structures by assessing RPCD, PRNFL thickness, and optic disc morphological parameters to detect subtle changes not apparent on routine ophthalmological examination.

## Materials and methods

This prospective study was conducted at a single center at Bakırköy Dr. Sadi Konuk Training and Research Hospital, in accordance with the principles of the Declaration of Helsinki and with approval from the local ethics committee (Approval Number: 2023/358). Informed consent was obtained from all participants.

From September to November 2023, 47 consecutive patients who underwent elective CPB surgery at the Cardiovascular Surgery Clinic were enrolled. After excluding two patients due to postoperative mortality, one patient with diabetic retinopathy, and four patients with inadequate OCT-A image quality, the final cohort consisted of included 40 patients (40 eyes).

CPB was performed using a coronary artery bypass machine with a hollow fiber membrane oxygenator and a 40 μm arterial filter. The ECC pump flow was maintained at 2.4 L/min/m² during aortic cross-clamping (ACC). Mean arterial pressure was maintained between 50 and 60 mmHg under moderate hypothermia (29–30 °C). Myocardial protection was achieved using intermittent antegrade cold blood cardioplegia. Intraoperative parameters, including total surgery duration, aortic cross-clamp time, ECC time, and intraoperative blood loss, were recorded for subsequent analysis.

All ophthalmological examinations and OCT-A measurements were performed at the Eye Clinic of Bakırköy Dr. Sadi Konuk Training and Research Hospital. Examinations included assessment of best-corrected visual acuity (BCVA), intraocular pressure (IOP), slit-lamp biomicroscopy, and dilated fundus examination. Baseline evaluations were performed within 3 days before surgery, and follow-up assessments were performed at postoperative week 1, week 2, and month 1.

Patients with a history of intraocular surgery, optic nerve disorders (including optic nerve drusen, myelinated nerve fibers, or glaucoma), orbital trauma, cranial pathology, or anterior or posterior segment conditions that could interfere with OCT-A image quality (such as advanced cataract, corneal opacity, vitreous opacity, or intravitreal hemorrhage) were excluded. Mild lens opacity was present in three patients (one with a best-corrected visual acuity of 8/10 and two with 9/10), all of whom had mild nuclear sclerosis. These opacities did not affect OCT-A image acquisition or quality.

Patients with diabetic or hypertensive retinopathy were also excluded. Only right eyes were included in the analysis. Eligible eyes had refractive errors within ± 2.00 diopters (spherical) and ± 1.50 diopters (cylindrical).

OCT-A imaging was performed using the RTVue-XR SD-OCT system (AngioVue; Optovue, Fremont, CA, USA). A 4.5 × 4.5 mm scan centered on the optic disc was obtained using standard peripapillary protocols. Each scan was automatically segmented using built-in software to visualize the peripapillary RNFL and RPCD. To ensure image quality, scans with a signal strength index below 6, obvious segmentation errors, or motion-related artifacts were excluded. All images were independently reviewed by two graders, and any discrepancies were resolved by consensus. All OCT-A measurements were acquired by the same technician between 09:00 12:00 to minimize diurnal variation.

### Statistical analysis

Statistical analyses were performed using the Statistical Package for the Social Sciences (SPSS) version 21.0 (IBM Corp., Armonk, NY, USA). Categorical variables were expressed as frequencies and percentages, while continuous variables were presented as mean ± standard deviation.

The normality of continuous variables was assessed using the Shapiro–Wilk test. Homogeneity of variances was evaluated using Levene’s test where applicable. The choice between parametric and non-parametric tests was based on the distribution characteristics of the data. Variables with a normal distribution were analyzed using parametric methods, whereas non-normally distributed variables were analyzed using non-parametric alternatives.

Given the repeated-measures design of the study, overall changes across time points were primarily evaluated using repeated measures ANOVA or the Friedman test, as appropriate. When appropriate, pairwise comparisons between baseline and follow-up measurements were performed using the paired samples t-test for normally distributed data and the Wilcoxon signed-rank test for non-normally distributed data.

Correlations between variables were assessed using Pearson or Spearman correlation coefficients according to data distribution.

In addition to statistical significance, effect sizes were calculated. Hedges’ g was used for parametric comparisons to account for small sample bias, while the rank-biserial correlation coefficient (r) was used for non-parametric comparisons. Correlation coefficients (Pearson r and Spearman rho) were interpreted as measures of effect size. For all effect size estimates, 95% confidence intervals were reported. All statistical analyses were conducted at a 95% confidence level, and *p* values < 0.05 were considered statistically significant.

A post hoc power analysis demonstrated a statistical power of 0.87, exceeding the conventionally accepted minimum threshold of 0.80 as recommended by Cohen (1988, Statistical Power Analysis for the Behavioral Sciences, 2nd ed., Lawrence Erlbaum Associates), suggesting that the study was adequately powered to detect clinically meaningful differences.

## Results

The study included 40 patients (40 eyes) with a mean age of 58.68 ± 11.63 years. Of these, 11 (27.5%) were female and 29 (72.5%) were male. Hypertension was present in 45% of patients, diabetes mellitus in 50%, both conditions in 27.5%, and chronic renal dysfunction in 7.5%. All patients had coronary artery disease. No patients showed evidence of diabetic retinopathy, hypertensive retinopathy, or optic neuropathy on preoperative or postoperative fundus examinations.

Preoperatively, all patients had a best-corrected visual acuity ≥ 8/10 (decimal notation) and an intraocular pressure < 20 mmHg. No pathological findings were detected in the optic disc or retinal examination. No changes were observed during the follow-up period.

The comorbid systemic conditions and intraoperative parameters are presented in Table 1.

**Table 1:** Preoperative comorbidities and intraoperative variables of patients undergoing cardiopulmonary bypass surgery (*n* = 40)

Comorbidity	Number of patients (*n*)
None	8
Diabetes Mellitus (DM)	20
Hypertension (HT)	18
DM + HT	3
Renal Dysfunctionᵃ	3
**Intraoperative variables**	**Mean ± SD**
Total surgery time (minutes)	320 ± 71
ACC time (minutes)	69 ± 28
ECC time (minutes)	130 ± 50
Blood loss (milliliters)	354 ± 218

Note: ᵃ Two patients with DM and 1 patient with HT. Data are presented as number of patients (n) for comorbidities and as mean ± standard deviation (SD) for intraoperative variables. n denotes the number of patients *Abbreviations*: ACC, aortic cross clamp; ECC, extracorporeal circulation

Preoperative and postoperative OCT-A measurements are summarized in Tables 2A, 2B, and 2C. No statistically significant differences were observed in RPCD, cup-to-disc (C/D) ratio, rim area, disc area, or cup volume at any postoperative time point (1 week, 2 weeks, or 1 month) compared with preoperative values.

**Fig. 1:**
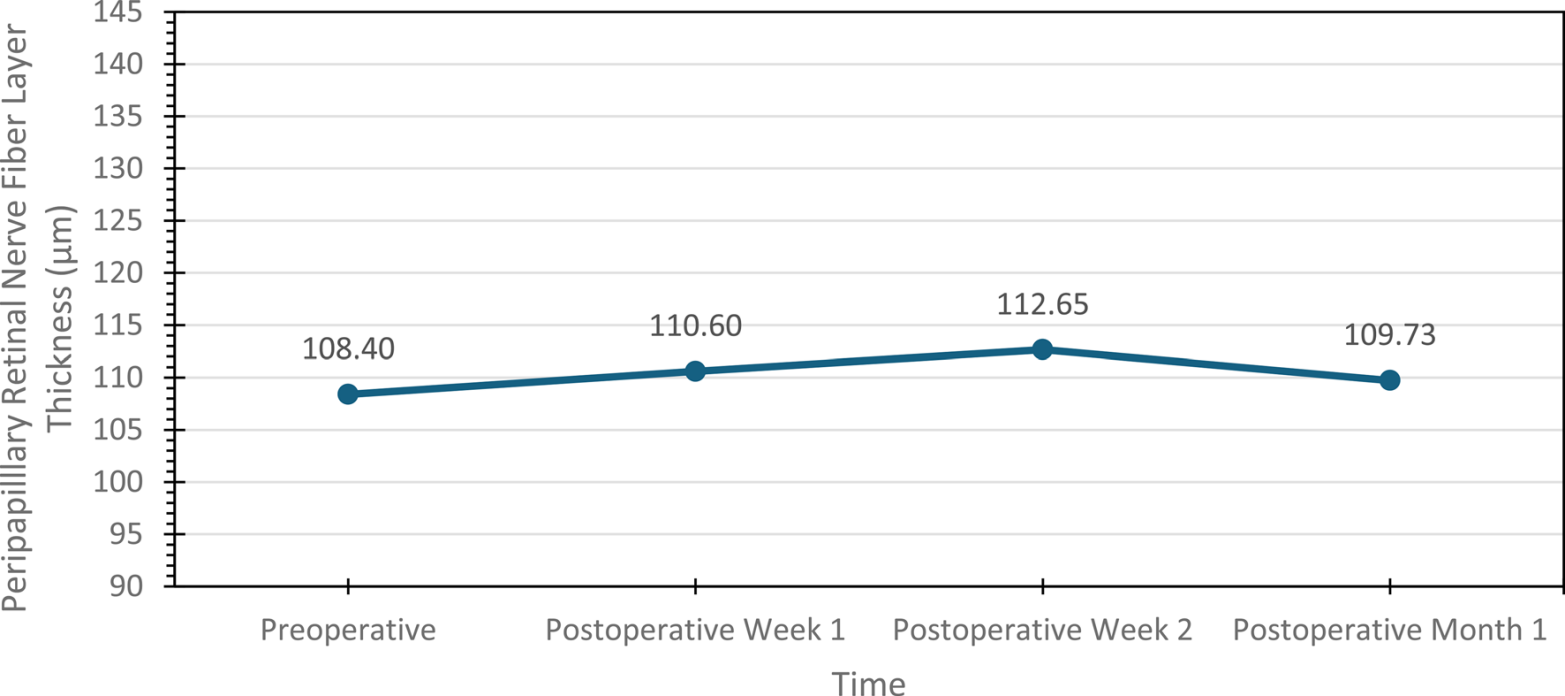
Changes in mean peripapillary retinal nerve fiber layer thickness over time

In contrast, PRNFL thickness increased significantly at postoperative weeks 1 and 2 compared with baseline (*p* = 0.0001 and *p* = 0.001, respectively). By postoperative month 1, PRNFL thickness returned to baseline values, with no statistically significant difference (Fig. 1).

**Table 2A:** Preoperative and postoperative week 1 OCT-A measurements of optic nerve head and peripapillary parameters (*n* = 40)

Parameter	Pre-op (Mean ± SD)	Post-op W1 (Mean ± SD)	*P*	Effect Size (95%CI)
RPCD (%)	57.88 ± 3.87	57.89 ± 4.14	0.820	0.04 (-0.313 to 0.384)
C/D horizontal	0.17 ± 0.20	0.20 ± 0.19	0.560	-0.147 (-0.563 to 0.327)
C/D vertical	0.20 ± 0.23	0.25 ± 0.25	0.146	-0.338 (-0.664 to 0.096)
Rim area (mm²)	1.82 ± 0.38	1.70 ± 0.35	0.220	0.225 (-0.135 to 0.533)
Disc area (mm²)	2.00 ± 0.48	1.89 ± 0.40	0.592	-0.273 (-0.273 to 0.43)
Cup volume (mm³)	0.03 ± 0.07	0.03 ± 0.04	0.457	-0.167 (-0.555 to -0.281)
PRNFL Thickness (µm)	108.40 ± 15.50	110.60 ± 15.22	**0.0001**	-0.677 (-0.832 to -0.425)

Note: Data are presented as mean ± standard deviation. P-values represent comparisons between each postoperative measurement and the corresponding preoperative value. A p-value < 0.05 was considered statistically significant. n denotes the number of patients *Abbreviations*: OCT-A, optical coherence tomography angiography; RPCD, radial peripapillary capillary density; C/D, cup-to-disc ratio; PRNFL, peripapillary retinal nerve fiber layer; mm², square millimeter; mm³, cubic millimeter; µm, micrometer; Pre-op, preoperative; Post-op, postoperative; W1, week 1; CI, confidence interval (95% CI represents the 95% confidence interval of the effect size)

A statistically significant change was observed in PRNFL thickness between the preoperative and postoperative week 1 measurements (*p* = 0.0001). The effect size was large (ES = -0.677), with a 95% confidence interval of -0.832 to -0.425. These findings suggest that the difference between the measurements is statistically significant and may indicate a strong effect size.

**Table 2B:** Preoperative and postoperative week 2 OCT-A measurements of optic nerve head and peripapillary parameters (*n* = 40)

Parameter	Pre-op (Mean ± SD)	Post-op W2 (Mean ± SD)	*P*	Effect Size (95%CI)
RPCD (%)	57.88 ± 3.87	57.14 ± 4.14	0.240	0.212 (-0.139 to 0.516)
C/D horizontal	0.17 ± 0.20	0.20 ± 0.20	0.630	-0.113 (-0.516 to 0.331)
C/D vertical	0.20 ± 0.23	0.23 ± 0.23	0.403	-0.196 (-0.582 to 0.262)
Rim area (mm²)	1.82 ± 0.38	1.76 ± 0.38	0.246	0.220 (-0.135 to 0.529)
Disc area (mm²)	2.00 ± 0.48	1.96 ± 0.49	0.245	0.222 (-0.148 to 0.537)
Cup volume (mm³)	0.03 ± 0.07	0.03 ± 0.07	0.830	0.06 (-0.378 to 0.476)
PRNFL Thickness (µm)	108.40 ± 15.50	112.65 ± 20.93	**0.001**	-0.611 (-0.788 to -0.341)

Note: Data are presented as mean ± standard deviation. P-values represent comparisons between each postoperative measurement and the corresponding preoperative value. A p-value < 0.05 was considered statistically significant. n denotes the number of patients *Abbreviations*: OCT-A, optical coherence tomography angiography; RPCD, radial peripapillary capillary density; C/D, cup-to-disc ratio; PRNFL, peripapillary retinal nerve fiber layer; mm², square millimeter; mm³, cubic millimeter; µm, micrometer; Pre-op, preoperative; Post-op, postoperative; W2, week 2; CI, confidence interval (95% CI represents the 95% confidence interval of the effect size)

A statistically significant change was observed in PRNFL thickness between the preoperative and postoperative week 2 measurements (*p* = 0.001). The effect size was large (ES = -0.611), with a 95% confidence interval of -0.788 to -0.341. These findings suggest that the difference between the measurements is statistically significant and may indicate a strong effect size.

**Table 2C:** Preoperative and postoperative month 1 OCT-A measurements of optic nerve head and peripapillary parameters (*n* = 40)

Parameter	Pre-op (Mean ± SD)	Post-op M1 (Mean ± SD)	*P*	Effect Size (95%CI)
RPCD (%)	57.88 ± 3.87	57.31 ± 4.02	0.180	0.245 (-0.11 to 0.544)
C/D horizontal	0.17 ± 0.20	0.18 ± 0.20	0.800	-0.06 (-0.476 to 0.378)
C/D vertical	0.20 ± 0.23	0.21 ± 0.23	0.903	-0.029 (-0.459 to 0.412)
Rim area (mm²)	1.82 ± 0.38	1.76 ± 0.42	0.696	0.073 (-0.279 to 0.408)
Disc area (mm²)	2.00 ± 0.48	1.93 ± 0.48	0.749	0.063 (-0.292 to 0.404)
Cup volume (mm³)	0.03 ± 0.07	0.03 ± 0.04	0.861	0.043 (-0.384 to 0.455)
PRNFL Thickness (µm)	108.40 ± 15.50	109.73 ± 19.80	0.276	-0.205 (-0.521 to 0.160)

Note: Data are presented as mean ± standard deviation. P-values represent comparisons between each postoperative measurement and the corresponding preoperative value. A p-value < 0.05 was considered statistically significant. n denotes the number of patients *Abbreviations*: OCT-A, optical coherence tomography angiography; RPCD, radial peripapillary capillary density; C/D, cup-to-disc ratio; PRNFL, peripapillary retinal nerve fiber layer; mm², square millimeter; mm³, cubic millimeter; µm, micrometer; Pre-op, preoperative; Post-op, postoperative; M1, month 1; CI, confidence interval (95% CI represents the 95% confidence interval of the effect size)

**Fig. 2:**
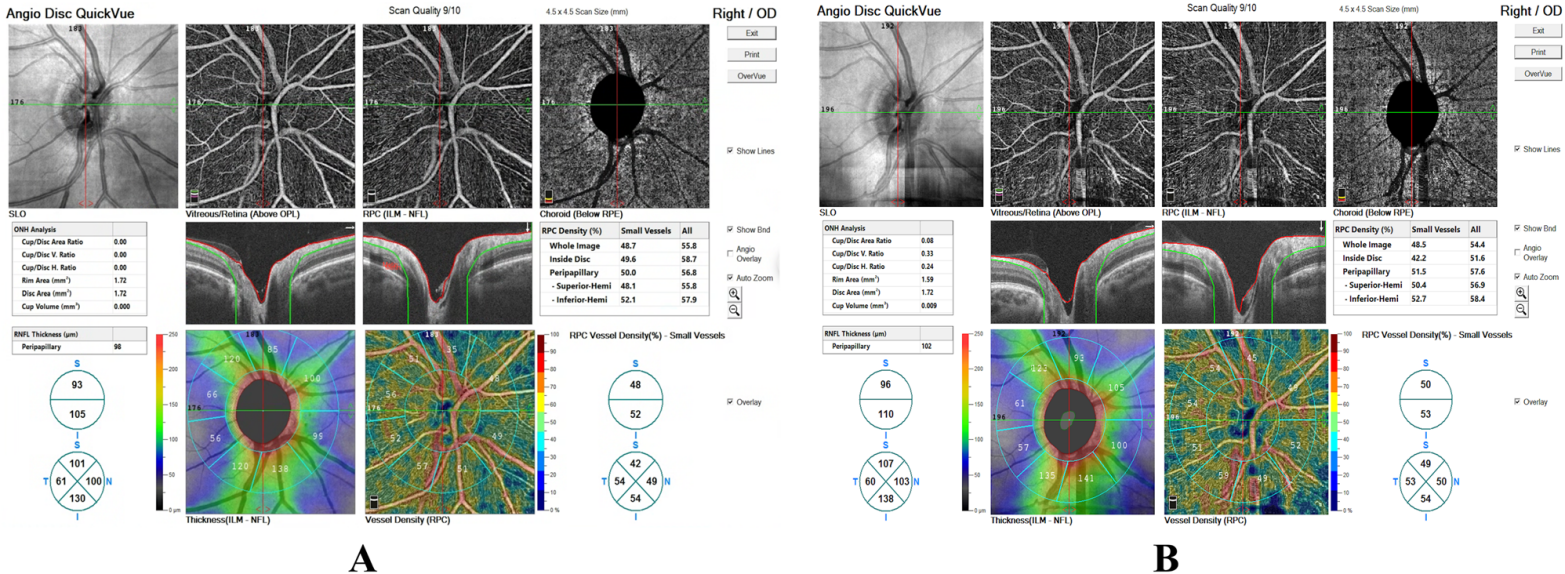
Representative OCT-A images of the optic nerve head before and after cardiopulmonary bypass surgery (**A**) Preoperative baseline. (**B**) Postoperative week 2

Representative OCT-A images obtained from a patient in the preoperative period and at postoperative week 2 are shown in Fig. 2.

Correlation analyses between OCT-A parameters and intraoperative variables are summarized in Tables 3, 4 and 5. At postoperative week 1, a moderate negative correlation was observed between ECC duration and between ECC duration and rim area change (*r* = − 0.432, *p* = 0.005), as well as between ACC time and rim area changes (*r* = − 0.343, *p* = 0.030). At postoperative week 2, total surgery duration was moderately negatively correlated with RPCD change (*r* = − 0.442, *p* = 0.005), while ECC (*r* = 0.405, *p* = 0.012) and ACC time (*r* = 0.479, *p* = 0.002) demonstrated moderate positive correlations with changes in cup volume. At postoperative month 1, a weak negative correlation was observed between ECC duration and rim area change (*r* = − 0.364, *p* = 0.021).

**Table 3:** Correlations between postoperative week 1 OCT-A parameters and intraoperative variables (*n* = 40)

Parameter	Total surgery time (min)	ECC time (min)	ACC time (min)	Blood loss (mL)
RPCD	*r* = -0.014 *p* = 0.933	*r* = 0.295 *p* = 0.069	*r* = 0.099 *p* = 0.548	*r* = 0.104 *p* = 0.528
C/D horizontal	*r* = -0.121 *p* = 0.458	*r* = 0.220 *p* = 0.172	*r* = 0.204 *p* = 0.207	*r* = -0.031 *p* = 0.847
C/D vertical	*r* = -0.042 *p* = 0.795	*r* = 0.195 *p* = 0.228	*r* = 0.195 *p* = 0.228	*r* = -0.122 *p* = 0.452
Rim area	*r* = -0.144 *p* = 0.375	*r* = -0.432 p = **0.005**	*r* = -0.343 p = **0.030**	*r* = 0.119 *p* = 0.465
Disc area	*r* = -0.178 *p* = 0.273	*r* = -0.288 *p* = 0.071	*r* = -0.224 *p* = 0.165	*r* = 0.076 *p* = 0.640
Cup volume	*r* = -0.134 *p* = 0.408	*r* = 0.172 *p* = 0.288	*r* = 0.147 *p* = 0.367	*r* = -0.084 *p* = 0.606
PRNFL Thickness	*r* = 0.083 *p* = 0.610	*r* = 0.281 *p* = 0.079	*r* = 0.197 *p* = 0.223	*r* = 0.220 *p* = 0.172

Note: Data are presented as mean ± standard deviation. Pearson correlation coefficient (r) was used for correlation analyses. Statistical significance was defined as *p* < 0.05. (*n* = 40). n denotes the number of patients *Abbreviations*: OCT-A, optical coherence tomography angiography; ECC, extracorporeal circulation; ACC, aortic cross clamp; RPCD, radial peripapillary capillary density; C/D, cup-to-disc ratio; PRNFL, peripapillary retinal nerve fiber layer

**Table 4:** Correlations between postoperative week 2 OCT-A parameters and intraoperative variables (*n* = 40)

Parameter	Total surgery time (min)	ECC time (min)	ACC time (min)	Blood loss (mL)
RPCD	*r* = -0.442 p = **0.005**	*r* = -0.145 *p* = 0.380	*r* = -0.296 *p* = 0.068	*r* = -0.011 *p* = 0.948
C/D horizontal	*r* = 0.014 *p* = 0.932	*r* = 0.223 *p* = 0.166	*r* = 0.268 *p* = 0.094	*r* = -0.044 *p* = 0.798
C/D vertical	*r* = 0.089 *p* = 0.585	*r* = 0.210 *p* = 0.192	*r* = 0.246 *p* = 0.126	*r* = -0.069 *p* = 0.674
Rim area	*r* = -0.009 *p* = 0.956	*r* = -0.416 *p* = 0.008	*r* = -0.257 *p* = 0.109	*r* = 0.081 *p* = 0.619
Disc area	*r* = -0.178 *p* = 0.273	*r* = -0.288 *p* = 0.071	*r* = -0.224 *p* = 0.165	*r* = 0.076 *p* = 0.640
Cup volume	*r* = 0.134 *p* = 0.410	*r* = 0.405 p = **0.012**	*r* = 0.479 p = **0.002**	*r* = -0.099 *p* = 0.544
PRNFL Thickness	*r* = -0.126 *p* = 0.437	*r* = -0.144 *p* = 0.374	*r* = -0.134 *p* = 0.409	*r* = 0.069 *p* = 0.674

Note: Data are presented as mean ± standard deviation. Pearson correlation coefficient (r) was used for correlation analyses. Statistical significance was defined as *p* < 0.05. (*n* = 40). n denotes the number of patients *Abbreviations*: OCT-A, optical coherence tomography angiography; ECC, extracorporeal circulation; ACC, aortic cross clamp; RPCD, radial peripapillary capillary density; C/D, cup-to-disc ratio; PRNFL, peripapillary retinal nerve fiber layer

**Table 5:** Correlations between postoperative month 1 OCT-A parameters and intraoperative variables (*n* = 40)

Parameter	Total surgery time (min)	ECC time (min)	ACC time (min)	Blood loss (mL)
RPCD	*r* = 0.005 *p* = 0.974	*r* = 0.025 *p* = 0.878	*r* = -0.100 *p* = 0.545	*r* = 0.180 *p* = 0.274
C/D horizontal	*r* = -0.132 *p* = 0.417	*r* = 0.195 *p* = 0.228	*r* = 0.159 *p* = 0.327	*r* = 0.056 *p* = 0.730
C/D vertical	*r* = -0.092 *p* = 0.571	*r* = 0.167 *p* = 0.303	*r* = 0.118 *p* = 0.470	*r* = 0.025 *p* = 0.880
Rim area	*r* = -0.208 *p* = 0.197	*r* = -0.364 p = **0.021**	*r* = -0.217 *p* = 0.178	*r* = -0.084 *p* = 0.607
Disc area	*r* = -0.040 *p* = 0.804	*r* = -0.254 *p* = 0.113	*r* = -0.073 *p* = 0.653	*r* = 0.237 *p* = 0.141
Cup volume	*r* = -0.143 *p* = 0.378	*r* = 0.137 *p* = 0.401	*r* = 0.092 *p* = 0.572	*r* = -0.017 *p* = 0.917
PRNFL Thickness	*r* = -0.049 *p* = 0.762	*r* = -0.123 *p* = 0.450	*r* = -0.114 *p* = 0.482	*r* = 0.076 *p* = 0.640

Note: Data are presented as mean ± standard deviation. Pearson correlation coefficient (r) was used for correlation analyses. Statistical significance was defined as *p* < 0.05. (*n* = 40). n denotes the number of patients *Abbreviations*: OCT-A, optical coherence tomography angiography; ECC, extracorporeal circulation; ACC, aortic cross clamp; RPCD, radial peripapillary capillary density; C/D, cup-to-disc ratio; PRNFL, peripapillary retinal nerve fiber layer

## Discussion

This study aimed to evaluate the early effects of CPB surgery on the peripapillary region using OCT-A, with a focus on changes in ONH morphological parameters, PRNFL thickness, and RPCD.

A transient increase in PRNFL thickness was detected at postoperative weeks 1 and 2, which returned to baseline levels by postoperative month 1. In contrast, no significant changes were detected in RPCD or optic disc morphological parameters, including the C/D ratio, rim area, disc area, and cup volume, during the follow-up period.

CPB involves the use of ECC to regulate venous return, cardiac output, and oxygenation, and this process may lead to systemic hemodynamic fluctuations. Considering its anatomical characteristics, the optic nerve head may be affected by these changes [8], and this mechanism may be associated with the early peripapillary findings observed in our study.

The autoregulatory mechanisms of the brain, retina, and ONH typically maintain blood flow within a critical perfusion pressure (PP) range of 50–150 mmHg [12, 13]. During CPB, particularly under moderate hypothermia, there is concern that ONH autoregulation may be compromised when PP falls below 50 mmHg, analogous to the loss of cerebral autoregulation [14, 15]. Nenekidis et al. [16] reported a significant reduction in optic nerve head blood flow during CPB, which was associated with the duration of ECC following CPB surgery; similarly, in a study of patients who underwent CPB using OCT-A, color Doppler imaging demonstrated impaired retrobulbar circulation [11].

The transient increase in PRNFL thickness observed in this study likely reflects a structural response to temporary hypoperfusion, most plausibly related to mild ischemic edema [17]. However, as direct measurements of ONH perfusion were not performed, this interpretation remains hypothesis-driven and exploratory, warranting further investigations incorporating direct perfusion assessments to elucidate the underlying mechanism.

This interpretation is consistent with the findings of Zeki Fikret C. et al. [17], who reported PRNFL thickening one week after CPB and attributed this change to ischemic edema. Similarly, studies on non-arteritic anterior ischemic optic neuropathy (NAION) have documented PRNFL thickening in the acute phase due to edema, followed by thinning in subsequent months [18, 19]. In this context, Roth and Moss [7] reported that ischemic optic neuropathy occurs in 0.06–0.113% of patients undergoing cardiac surgery and that, in some cases, optic disc edema resolves over weeks to months, subsequently progressing to optic atrophy. Our study was not designed to detect such rare clinical events, but rather to identify early structural and microvascular changes detectable by OCT-A.

On the other hand, in one of the limited studies examining ONH changes using OCT-A in patients undergoing CPB, Gabija Vicaite et al. [11] reported no significant change in PRNFL thickness compared with a control group, while Büyükateş et al. [20] reported a decrease in PRNFL thickness during the first postoperative month following CPB surgery. When these differing findings are taken into consideration, our results suggest the presence of an initial edematous phase that gradually resolves and may potentially mask subsequent thinning that could become apparent with longer follow-up.

With respect to vascular parameters, no statistically significant differences were observed in RPCD between preoperative and postoperative measurements. This finding is consistent with the results of Şimdivar et al. [21], who similarly reported no significant changes in peripapillary vessel density at two weeks following CPB. In contrast, Gabija et al. [11] reported reduced vascular density in patients undergoing CPB compared with healthy controls; however, unlike their study, our analysis was based on within-subject comparisons using each patient’s own preoperative baseline. In a different clinical context, Rebolleda et al. [22] reported a decrease in RPCD within the first three months following resolution of optic disc edema in patients with non-arteritic anterior ischemic optic neuropathy. The absence of significant changes in RPCD in our cohort suggests that, despite intraoperative hemodynamic and inflammatory stress, the ONH microvasculature may be capable of maintaining capillary perfusion in the early postoperative period through autoregulatory mechanisms. This interpretation is further supported by OCT-based evidence indicating early postoperative hemodynamic stabilization [23].

Nevertheless, the moderate negative correlation identified between total surgery duration and RPCD at postoperative week 2 suggests that prolonged operative stress may exert a subtle, time-dependent effect on optic nerve perfusion that is not captured by group-level comparisons alone. In addition to RPCD, correlation analyses indicated moderate relationships between intraoperative variables and certain optic disc structural parameters. At postoperative week 1, longer ECC and ACC durations were moderately associated with reductions in rim area, while at postoperative week 2, ECC and ACC times showed moderate positive correlations with changes in cup volume. By postoperative month 1, a weak negative correlation was observed between ECC duration and rim area change. However, that a relatively large number of correlations were performed across multiple parameters and time points without formal correction for multiple comparisons. Therefore, the possibility of type I error cannot be excluded. These findings should be interpreted with caution and considered exploratory and hypothesis-generating rather than confirmatory. Overall, the observed correlations may suggest subtle, time-dependent effects of intraoperative factors on peripapillary structural and microvascular parameters; however, their clinical significance remains uncertain, and further studies with larger sample sizes and appropriate adjustment for multiple testing are warranted to validate these preliminary observations.

Taken together, our RPCD findings indicate that CPB does not result in a measurable early postoperative reduction in peripapillary capillary density; however, the observed association between longer surgical duration and reduced RPCD at postoperative week 2 suggests that operative time–related stress may influence ONH microcirculation in a subtle and time-dependent manner.

In this study, no statistically significant differences were observed in optic disc morphological parameters, including the C/D ratio, rim area, disc area, and cup volume, between preoperative and postoperative measurements. This finding is consistent with the results reported by Zeki Fikret C. et al. [17], who similarly found no significant changes in optic disc cup volume or C/D ratio one week after CPB surgery. In contrast, Gabija et al. [11] reported a significantly smaller rim area and a higher vertical C/D ratio in patients undergoing CPB compared with healthy controls. The discrepancy between our findings and those of Gabija et al. may be attributable to differences in study design, as our analysis was based on within-subject comparisons between preoperative and postoperative OCT-A measurements rather than comparisons with a healthy control group.

A potential concern when interpreting non-significant findings is whether the study had sufficient statistical power to detect meaningful differences. In this study, a post hoc power analysis demonstrated a statistical power of 0.87, exceeding the commonly accepted threshold of 0.80, suggesting that the sample size was generally adequate to detect clinically relevant differences. Therefore, the absence of statistically significant changes in vascular parameters such as RPCD should be interpreted cautiously and may not necessarily reflect a true lack of effect. Nonetheless, the possibility that small or subtle microvascular changes were not detected cannot be excluded. Furthermore, considering the relatively low incidence of severe ischemic complications following cardiopulmonary bypass surgery, these findings should be interpreted with caution and may reflect early subclinical changes.

The systemic comorbidities of the participants were well controlled and remained clinically stable throughout the study period. None of the patients developed an acute or decompensated condition, and no systemic disease fluctuations that could have significantly influenced OCT-A measurements were observed. Nevertheless, considering that our cohort included patients with systemic comorbidities such as Diabetes Mellitus and Hypertension, their potential confounding effects on retinal microvascular parameters cannot be completely excluded.

Previous OCT-A studies have demonstrated that both diabetes and hypertension are associated with alterations in retinal capillary density and microvascular architecture [24,25]. However, these changes are generally considered to reflect chronic cumulative vascular damage rather than short-term fluctuations. Indeed, even in the absence of clinically visible retinopathy, microvascular alterations in diabetes have been shown to develop over long disease durations, and longitudinal studies indicate that such changes tend to evolve gradually over time rather than within short follow-up intervals [26, 27].

Therefore, although the lack of multivariate adjustment or subgroup analyses is a limitation of the present study, the short follow-up period and the clinical stability of systemic conditions suggest that the influence of these comorbidities on the observed postoperative changes is likely limited. Future studies with larger sample sizes should incorporate adjusted statistical models to better elucidate the independent effects of cardiopulmonary bypass on ocular microcirculation.

Although mild lens opacity was present in a small number of patients, all cases were limited to mild nuclear sclerosis and did not affect image quality. Furthermore, as OCT-A measurements were performed longitudinally in the same individuals, any minimal effect of lens opacity would have been consistent across time points and therefore unlikely to influence the observed changes.

Our study has several limitations. Firstly, the relatively small sample size and the short follow-up period of one month limit the generalizability of our findings and preclude observation of long-term changes, such as late-onset optic nerve atrophy reported in cases of ischemic optic neuropathy [7]. Although the systemic comorbidities of the participants remained stable throughout the study period, their potential influence on the observed outcomes cannot be entirely excluded. Additionally, visual field testing, which functional correlations to the structural changes observed, was not included in our study. This omission limits the clinical interpretation of the structural findings.

In conclusion, this study suggests that CPB surgery is associated with early but reversible changes in the ONH and peripapillary retinal structures.However, the absence of functional correlation limits the ability to determine the clinical significance of these structural changes. Therefore, these findings should be considered exploratory. Long-term studies are needed to determine whether these early structural changes progress to permanent axonal damage, and further research, including functional assessments, is necessary to clarify their clinical significance.

## Data Availability

The datasets generated and/or analyzed during the current study are available from the corresponding author on reasonable request, subject to institutional and ethical regulations regarding patient confidentiality.
